# Polymorphisms in *NFKB1* and *TLR4* and Interaction with Dietary and Life Style Factors in Relation to Colorectal Cancer in a Danish Prospective Case-Cohort Study

**DOI:** 10.1371/journal.pone.0116394

**Published:** 2015-02-23

**Authors:** Tine Iskov Kopp, Vibeke Andersen, Anne Tjonneland, Ulla Vogel

**Affiliations:** 1 National Food Institute, Technical University of Denmark, 2860 Søborg, Denmark; 2 Organ Center, Hospital of Southern Jutland, 6200 Aabenraa, Denmark; 3 Institute of Regional Health Research, University of Southern Denmark, 5000 Odense, Denmark; 4 Medical Department, Regional Hospital Viborg, 8800 Viborg, Denmark; 5 Danish Cancer Society Research Center, 2100 Copenhagen, Denmark; 6 National Research Centre for the Working Environment, 2100 Copenhagen, Denmark; National Health Research Institutes, TAIWAN

## Abstract

Maintenance of a balance between commensal bacteria and the mucosal immune system is crucial and intestinal dysbiosis may be a key event in the pathogenesis of colorectal cancer (CRC). The toll-like receptor 4 (TLR4) is an important pattern-recognition receptor that regulates inflammation and barrier function in the gut by a mechanism that involves activation of the nuclear factor–κB (NF-κB) transcription factor. Dietary and life style factors may impact these functions. We therefore used a Danish prospective case-cohort study of 1010 CRC cases and 1829 randomly selected participants from the Danish Diet, Cancer and Health cohort to investigate three polymorphisms in *NFKB1* and *TLR4* and their possible interactions with diet and life style factors in relation to risk of CRC. Homozygous carriage of the variant allele of the *TLR4*/rs5030728 polymorphism was associated with increased risk of CRC (incidence rate ratio (IRR) = 1.30; 95% confidence interval (CI): 1.05–1.60; P = 0.02 (gene-dose model); IRR = 1.24; 95%CI: 1.01–1.51; P = 0.04 (recessive model)). Del-carriers of the *NFKB1*/rs28362491 polymorphism had a 17% (95%CI: 1.03–1.34; P = 0.02) increased risk of CRC compared to homozygous carriers of the ins-allele. However, none of these risk estimates withstood adjustment for multiple comparisons. We found no strong gene-environment interactions between the examined polymorphism and diet and life style factors in relation to CRC risk.

## Introduction

Colorectal cancer (CRC) is the third most common cancer type in men and the second in women worldwide [1]. Hereditary factors are estimated to contribute to only 35% of the risk [2] emphasizing the importance of environmental factors in the etiology of CRC. Indeed, dietary and lifestyle factors have been intensively studied for their role in colorectal carcinogenesis; and alcohol, smoking, obesity and high meat intake are now established risk factors for CRC [3–5]. Intake of red and processed meat has the potential of inducing cancer by chemical carcinogens formed during cooking of meat at high temperatures [6], production of toxic fermentation compounds [7–9] or by inducing inflammation due to changes in bacterial composition [10]. Conversely, fibre, fruit and vegetables provide short-chain fatty acids (SCFAs) to the colonic epithelium by fermentation of unabsorbed dietary fibre and starch [11–15]. The SCFA butyrate is important for colonic integrity [14], inhibits growth of cancer cells *in vitro* [16,17] and has anti-inflammatory properties mainly by the inhibition of nuclear factor—κB (NF-κB) activation [18].

Toll-like receptors (TLR) are important pattern-recognition receptors that regulate inflammation and barrier function in the gut thereby maintaining a balance between commensal bacteria and the mucosal immune system [19,20]. The receptor for gram-negative bacterial lipopolysaccharide (LPS), TLR4, regulates cell proliferation in response to cell injury through induction of cyclooxygenase 2 expression [21] in a cascade that involves activation of NF-κB and epidermal growth factor [22] suggesting that TLR4 is an important element in the transition from inflammation to neoplasia [19]. Indeed, increased expression of TLR4 has been linked to development of inflammation-associated neoplasia [23–26]. In addition, we have previously found evidence that inflammation may contribute to CRC carcinogenesis. Thus, genetically determined high IL-1β and COX-2 levels were associated with increased risk of CRC [27].

Dysbiosis in the gut may be a key event in the pathogenesis of both inflammatory bowel diseases (IBD) and CRC. Using functional single nucleotide polymorphisms (SNPs) and their interaction with diet and life style may reveal important pathways for colorectal carcinogenesis [28]. Since *TLR* polymorphisms have been associated with IBD [29], we aimed to examine a possible mutual mechanism for IBD and CRC.

We have previously shown that carriage of the variant del-allele of the functional ins/del *NFKB1*/rs28362491 polymorphism is associated with increased risk of CRC and interacted with meat intake in a subset of the current study group [30] in agreement with results from two other studies also reporting increased risk of CRC among variant carriers of the polymorphism among a Malaysian [31] and a Swedish population [32], but not a Chinese study group [32]. A functional SNP in *TLR4* (rs4986790) has been extensively studied and has been associated with neoplastic progression *in vitro* [33], aggressive human colon cancer [33], IBD [34–37] and CRC [38–40]. However others were not able to find an association between CRC and the SNP [41–43]. In a Canadian study [44], intake of dietary saturated fatty acids was inversely related to blood level of high density lipoprotein cholesterol in individuals homozygous for the *TLR4*/rs5030728 G-allele. *TLR4*/rs5030728 may therefore interact with dietary components in the gut.

Thus, we expected that inflammation is an important factor in colorectal carcinogenesis and thus examined whether diet and life style factors (non-steroidal anti-inflammatory drugs (NSAID) and smoking) modify CRC risk by altering the mucosal immune response in the gut via interacting with TLR4 and NF-κB. We therefore evaluated three polymorphisms in *NFKB1* and *TLR4* and their possible interaction with diet and life style factors in a prospective cohort of 1010 CRC cases and 1829 randomly selected participants from the Danish Diet, Cancer and Health Study.

## Material and Methods

### Studied subjects

The Diet, Cancer and Health study is an ongoing Danish cohort study designed to investigate the relation between diet, lifestyle and cancer risk [45]. The cohort consists of 57,053 persons, recruited between December 1993 and May 1997. All the subjects were born in Denmark, and the individuals were 50 to 64 years of age and had no previous cancers at study entry. Blood samples and questionnaire data on diet and lifestyle were collected at study entry.

### Follow-up and endpoints

Follow-up was based on population-based cancer registries. Between 1994 and 31th December 2009, 1010 CRC cases were diagnosed. A sub-cohort of 1829 persons was randomly selected within the cohort. 28 persons were both cases and sub-cohort due to the used study design [46]. 245 with missing genotype data and 16 with missing data on risk factors were excluded. All information on genotypes and diet and lifestyle factors was available for 915 CRC cases and 1719 sub-cohort members.

### Dietary and lifestyle questionnaire

Information on diet, lifestyle, weight, height, medical treatment, environmental exposures, and other socio-economic factors were collected at enrolment using questionnaires and interviews and has been described in details elsewhere [27,47–49]. In short, the food-frequency questionnaire, diet consumption was assessed in 12 categories of predefined responses, ranking from ‘never’ to ‘eight times or more per day’. The daily intake was then calculated by FoodCalc [45]. Smoking status was classified as never, past or current. Persons smoking at least 1 cigarette daily during the last year were classified as smokers. NSAID use (“Aspirin”, “Paracetamol”, “Ibuprofen”, or “Other pain relievers) was assessed as ≥ 2 pills per month during one year at baseline.

### Genotyping

Buffy coat preparations were stored at minus 150°C until use. DNA was extracted as described [50]. The DNA was genotyped by LGC KBioscience (LGC KBioscience, Hoddesdon, United Kingdom) by PCR-based KASP genotyping assay (**Error! Hyperlink reference not valid**. www.lgcgenomics.com/). *NFKB1*/rs28362491 was analysed and reported for a subset of the current study group [30]. Two of the polymorphisms (*NFKB1*/rs28362491 and *TLR4*/rs4986790) were chosen based on known functionality and their association with CRC [30–33] and IBD [34–37] from a literature search. The *TLR4*/rs5030728 polymorphism, on the other hand, has no known functionality. However, *TLR4*/rs4986790 is tightly linked with *TLR4*/rs5030728 (D’: 1.0; r^2^: 0.017) using Haploview version 4.2 (Broad Institute of MIT and Harvard, Cambridge) [51] with HapMap3 Genome Browser release #2 (Phase 3) [52]; and since *TLR4*/rs5030728 has a higher minor allele frequency in Caucasians than *TLR4*/rs4986790 (0.305 vs. 0.035), this polymorphism is more suitable for gene-environment interaction analyses. To confirm reproducibility, genotyping was repeated for 10% of the samples yielding 100% identity.

### Statistical analysis

Deviation from Hardy-Weinberg equilibrium was assessed using a Chi-square test.

Incidence rate ratios (IRR) and 95% Confidence Interval (CI) were calculated according to the principles for analysis of case-cohort studies using an un-weighted approach [46]. Age was used as the time scale in the Cox regression models. Tests and confidence intervals were based on Wald’s tests using the robust estimate of the variance-covariance matrix for the regression parameters in the Cox regression models [53] as previously described [27,46,48,54–61].

All models were adjusted for baseline values of risk factors for colorectal cancer such as body mass index (BMI) (kg/m^2^, continuous), use of hormone replacement therapy (HRT) (never/past/current, among women), intake of dietary fibre (g/day, continuous), and red meat and processed meat (g/day, continuous) and in addition to suspected risk factors such as NSAID use (yes/no) and smoking status (never/past/current). Cereals, fibre, fruit and vegetables were also entered linearly. All analyses were stratified by gender, so that the basic (underlying) hazards were gender specific. For all the polymorphisms, IRR was calculated separately for heterozygous and homozygous variant allele carriers. For *TLR4*/rs4986790 and *NFKB1*/rs28362491, variant allele carriers were subsequently grouped for interaction analyses since no recessive effects were observed. *TLR4/*rs5030728 was inferred both in a gene-dose and a recessive mode in the subsequent analyses.

Moreover, we assessed weekly use of NSAID based on the results of a study of colorectal cancer within the Diet, Cancer and Health cohort [62] reporting that long-term consistent use of Aspirin or Non-Aspirin NSAID appears necessary to achieve a protective effect. However, there were no differences in risk estimates between monthly or weekly use, consequently, to maintain the statistical power in the strata; we used monthly NSAID use in the analyses.

The likelihood ratio test was used for interaction analyses between the studied polymorphisms and intake of red and processed meat, dietary fibre, cereals, fish, fruits, vegetables, alcohol intake, smoking status and NSAID use. In interaction analyses where the dietary factors were entered as categorical variables, tertile cutpoints were based on the empirical distribution among male and female cases, respectively. The possible interactions were investigated using the likelihood ratio test.

All analyses were performed using SAS version 9.3 (SAS Institute Inc., Cary, NC). A p<0.05 was considered to be significant. Moreover, to test for multiple comparisons, Bonferroni correction was used.

### Ethics statement

All participants gave verbal and written informed consent. The Diet, Cancer and Health study was approved by the National Committee on Health Research Ethics (journal nr. (KF) 01–345/93) and the Danish Data Protection Agency.

## Results

Baseline characteristics of the study population are presented in Table 1. Among sub-cohort members, the genotype distribution of the SNPs did not deviate from Hardy-Weinberg equilibrium (results not shown).

**Table 1:** Baseline characteristics of the study participants by selected demographic and established CRC risk factors.

Variable	Cases		Sub-cohort		IRR^a^ (95% CI)
	n (%)	Median (5–95%)	n (%)	Median (5–95%)	
Total	915 (100)		1719 (100)		
Sex					
Men	515 (56)		920 (54)		
Women	400 (44)		799 (46)		
Age at inclusion (years)		58 (51–64)		56 (50–64)	
BMI (kg/m^2^)		26.3 (20.7–34.3)		25.6 (20.5–33.0)	1.03 (1.00–1.06)^d^
Food intake (g/day)					
Alcohol^b^		15.1 (1.0–71.6)		14.2 (1.2–65.3)	1.03 (1.00–1.07)^e^
Dietary fibre		19.9 (10.8–32.9)		20.7 (10.7–34.2)	0.88 (0.80–0.97)^f^
Red and processed meat		113.1 (47.4–233.4)		108.9 (41.5–235.4)	1.03 (1.00–1.06)^g^
Smoking status					
Never	274 (30)		572 (33)		1.00 (ref.)
Past	280 (31)		513 (30)		1.04 (0.88–1.23)
Current	361 (39)		634 (37)		1.11 (0.94–1.30)
NSAID use^c^					
No	632 (69)		1174 (68)		1.00 (ref.)
Yes	283 (31)		545 (32)		0.99 (0.86–1.14)
HRT use among women					
Never	246 (62)		418 (52)		1.00 (ref.)
Past	50 (13)		126 (16)		0.66 (0.49–0.90)
Current	104 (26)		255 (32)		0.74 (0.59–0.93)

Values are expressed as medians (5th and 95th percentiles) or as fractions (%).

^a^IRRs for CRC—mutually adjusted.

^b^Among current drinkers.

^c^NSAID use is defined as ≥ 2 pills per month during one year.

^d^Risk estimate per 2 kg/m^2^ increment of BMI.

^e^Risk estimate for the increment of 10 g alcohol per day.

^f^Risk estimate for the increment of 10 g dietary fibres per day.

^g^Risk estimate for the increment of 25 g red and processed meat per day.

### Associations between polymorphisms and CRC

Homozygous variant carriers of the *TLR4*/rs5030728 polymorphism were at 1.30-fold (95%CI: 1.05–1.60) increased risk of CRC in a gene-dose model and at 1.24-fold (95%CI: 1.01–1.51) increased risk of CRC compared to wild type and heterozygous carriers in a recessive model (Table 2). Moreover, carriers of the *NFKB1* del-allele had a 17% (95%CI: 1.03–1.34) increased risk of CRC compared to homozygous carriers of the ins-allele (Table 2). These risk estimates did not, however, reach statistically significance after Bonferroni correction. There was no interaction between the two risk genotypes *TLR4*/rs5030728 and *NFKB1*/rs28362491 but on the other hand, there was no additive effect of being homozygous carrier of both variant alleles (S1 Table).

**Table 2:** IRR for CRC in relation to the studied polymorphisms.

		n_cases_ (%)	n_sub-cohort_ (%)	IRR^a^ (95% CI)	IRR^b^ (95% CI)	*P*-value^c^
***TLR4***	rs4986790					
	AA	839 (92)	1577 (92)	1.00 (ref.)	1.00 (ref.)	-
	GA	76 (8)	141 (8)	0.99 (0.79–1.25)	1.00 (0.79–1.26)	0.98
	GG	0 (0)	1 (0)	-	-	-
	GA+GG	76 (8)	142 (8)	0.99 (0.79–1.25)	1.00 (0.79–1.26)	0.97
	rs5030728					
	GG	405 (44)	826 (48)	1.00 (ref.)	1.00 (ref.)	-
	GA	399 (44)	731 (43)	1.10 (0.96–1.26)	1.11 (0.96–1.27)	0.16
	AA	111 (12)	162 (9)	1.30 (1.06–1.61)	1.30 (1.05–1.60)	0.02
	GA+AA	510 (56)	398 (23)	1.14 (1.00–1.30)	1.14 (1.00–1.30)	0.05
	AA vs. GG+GA	111 (12)	162 (9)	1.24 (1.02–1.52)	1.24 (1.01–1.51)	0.04
***NFKB1***	rs28362491					
	Ins/Ins	320 (35)	679 (60)	1.00 (ref.)	1.00 (ref.)	-
	Ins/Del	449 (49)	787 (46)	1.19 (1.03–1.37)	1.19 (1.03–1.37)	0.02
	Del/Del	146 (16)	253 (15)	1.13 (0.93–1.37)	1.14 (0.94–1.38)	0.18
	Ins/Del+Del/Del	595 (65)	1040 (61)	1.17 (1.02–1.34)	1.17 (1.03–1.34)	0.02

^a^ Crude—adjusted for age and sex.

^b^ In addition, adjusted for smoking status, alcohol intake, HRT status (women only), BMI, use of NSAID, intake of red and processed meat, and dietary fibre.

^c^ P-value for the adjusted estimates.

### Gene-environment analyses

We found no interaction between any of the dietary factors and the studied polymorphisms in relation to risk of CRC in the linear analyses (S2 Table). In the tertile analyses, there was weak interaction between *TLR4*/rs5030728 and intake of vegetables (Gene-dose model: P-value for interaction (P_int_) = 0.05; Recessive model: P_int_ = 0.03) (Table 3). Moreover, a borderline statistically significant interaction between meat intake and the *NFKB1*/rs28362491 polymorphism (P_int_ = 0.06) was found (Table 3). For ins-carriers, risk estimates were comparable across tertiles of meat intake. Conversely, among del-carriers, intake of meat in the second (IRR = 1.46; 95%CI: 1.17–1.83) and third tertile (IRR = 1.24; 95%CI: 0.99–1.56) was associated with risk of CRC, whereas del-allele carriers were not at risk in the first tertile with low meat intake (IRR = 0.98; 95%CI: 0.78–1.23). With regard to alcohol, variant carriers of *NFKB1*/rs28362491 and homozygous A-allele carriers of *TLR4*/rs5030728 were associated with CRC risk compared to the homozygous wild type carriers among participants with a low intake of alcohol. Furthermore, for variant carriers of all three polymorphisms, a low intake (first tertile) of alcohol was associated with the highest CRC risk compared with moderate (second tertile) intake—which was associated with the lowest risk—and high alcohol intake (third tertile) (Table 3).

**Table 3:** IRR for CRC for tertiles of intake of dietary factors for the studied polymorphisms.

	1.tertile		2.tertile		3.tertile		1.tertile	2.tertile	3.tertile	*P*-value^b^	1.tertile		2.tertile		3.tertile		1.tertile	2.tertile	3.tertile	*P*-value^b^
	Nc	Ns	Nc	Ns	Nc	Ns	IRR (95%CI)^a^	IRR (95%CI) ^a^	IRR (95%CI) ^a^		Nc	Ns	Nc	Ns	Nc	Ns	IRR (95%CI) ^a^	IRR (95%CI) ^a^	IRR (95%CI) ^a^	
*TLR4*/rs4986790							***Red and processed meat***										***Fish***			
AA	273	598	248	457	288	522	1.00 (ref.)	1.31 (1.11–1.55)	1.22 (1.04–1.54)		279	534	286	496	274	547	1.00 (ref.)	1.02 (0.86–1.20)	0.92 (0.77–1.10)	
GA+GG	29	49	27	48	20	45	1.15 (0.77–1.72)	1.22 (0.85–1.76)	0.98 (0.62–1.55)	0.49	27	48	21	46	28	48	1.06 (0.70–1.60)	0.85 (0.55–1.32)	0.93 (0.63–1.37)	0.72
	***Dietary cereal***																***Dietary fibre***			
AA	282	445	277	534	280	598	1.00 (ref.)	0.88 (0.74–1.05)	0.94 (0.75–1.17)		279	479	277	464	283	634	1.00 (ref.)	0.99 (0.84–1.16)	0.81 (0.69–0.96)	
GA+GG	19	44	30	42	27	56	0.74 (0.46–1.19)	1.01 (0.69–1.47)	0.96 (0.64–1.46)	0.33	21	48	29	36	26	58	0.78 (0.49–1.22)	1.29 (0.88–1.90)	0.72 (0.49–1.07)	0.19
	***Fruit***																***Vegetables***			
AA	275	487	287	535	277	555	1.00 (ref.)	0.96 (0.81–1.14)	0.95 (0.77–1.16)		275	466	278	549	286	562	1.00 (ref.)	1.02 (0.86–1.21)	1.08 (0.89–1.32)	
GA+GG	25	39	22	52	29	51	1.04 (0.69–1.56)	0.81 (0.52–1.27)	0.97 (0.66–1.44)	0.75	28	34	26	59	22	49	1.17 (0.79–1.72)	0.91 (0.60–1.38)	0.95 (0.62–1.47)	0.55
	***Alcohol***																			
AA	288	517	277	578	274	482	1.00 (ref.)	0.92 (0.78–1.08)	1.10 (0.93–1.31)											
GA+GG	33	42	22	53	21	47	1.23 (0.86–1.78)	0.80 (0.52–1.22)	0.89 (0.57–1.40)	0.28										
*TLR4*/rs5030728							***Red and processed meat***										***Fish***			
GG	134	295	134	255	137	276	1.00 (ref.)	1.19 (0.94–1.51)	1.08 (0.86–1.36)		127	284	139	262	139	280	1.00 (ref.)	1.08 (0.86–1.37)	1.00 (0.78–1.27)	
GA	133	284	134	210	132	237	1.00 (0.79–1.27)	1.34 (1.06–1.69)	1.26 (1.00–1.60)		137	240	131	227	131	264	1.21 (0.94–1.54)	1.13 (0.89–1.44)	1.03 (0.81–1.32)	
AA	35	68	37	40	39	54	1.06 (0.73–1.55)	1.70 (1.19–2.43)	1.54 (1.09–2.17)	0.73	42	58	37	53	32	51	1.39 (0.99–1.96)	1.32 (0.92–1.90)	1.23 (0.83–1.81)	0.90
GG+GA	267	579	268	465	269	513	1.00 (ref.)	1.26 (1.06–1.49)	1.16 (0.98–1.37)		264	524	270	489	270	544	1.00 (ref.)	1.01 (0.85–1.20)	0.93 (0.78–1.11)	
AA	35	68	37	40	39	54	1.06 (0.74–1.52)	1.70 (1.21–2.38)	1.54 (1.11–2.13)	0.55	42	58	37	53	32	51	1.27 (0.92–1.75)	1.20 (0.85–1.70)	1.12 (0.77–1.63)	0.96
							***Dietary cereal***										***Dietary fibre***			
GG	125	233	136	275	144	318	1.00 (ref.)	0.97 (0.76–1.24)	1.00 (0.76–1.31)		130	254	131	234	144	338	1.00 (ref.)	1.05 (0.83–1.33)	0.83 (0.66–1.05)	
GA	126	211	145	244	128	276	1.10 (0.85–1.41)	1.07 (0.84–1.37)	1.07 (0.81–1.42)		126	223	142	217	131	291	1.10 (0.86–1.40)	1.14 (0.91–1.44)	0.89 (0.70–1.14)	
AA	50	45	26	57	35	60	1.54 (1.12–2.13)	0.89 (0.58–1.36)	1.34 (0.90–2.00)	0.34	44	50	33	49	34	63	1.35 (0.96–1.90)	1.22 (0.84–1.78)	1.11 (0.76–1.62)	0.98
GG+GA	251	444	281	519	272	594	1.00 (ref.)	0.97 (0.81–1.17)	0.99 (0.79–1.23)		256	477	273	451	275	629	1.00 (ref.)	1.05 (0.89–1.24)	0.82 (0.69–0.98)	
AA	50	45	26	57	35	60	1.48 (1.09–1.99)	0.85 (0.57–1.28)	1.28 (0.88–1.87)	0.10	44	50	33	49	34	63	1.30 (0.94–1.78)	1.17 (0.82–1.67)	1.06 (0.74–1.52)	0.79
							***Fruit***										***Vegetables***			
GG	139	264	135	275	131	287	1.00 (ref.)	0.98 (0.77–1.24)	0.91 (0.70–1.18)		132	250	130	289	143	287	1.00 (ref.)	1.03 (0.80–1.31)	1.16 (0.90–1.50)	
GA	126	212	136	260	137	259	1.10 (0.87–1.40)	0.98 (0.78–1.25)	1.07 (0.83–1.39)		136	187	129	269	134	275	1.28 (1.01–1.62)	1.06 (0.83–1.35)	1.15 (0.88–1.50)	
AA	35	50	38	52	38	60	1.26 (0.88–1.82)	1.24 (0.87–1.76)	1.21 (0.83–1.75)	0.92	35	63	45	50	31	49	1.02 (0.70–1.47)	1.75 (1.26–2.44)	1.44 (0.96–2.15)	0.05
GG+GA	265	476	271	353	268	546	1.00 (ref.)	0.94 (0.79–1.12)	0.94 (0.77–1.15)		268	437	259	558	277	562	1.00 (ref.)	0.93 (0.78–1.11)	1.03 (0.84–1.26)	
AA	35	50	38	52	38	60	1.21 (0.85–1.71)	1.18 (0.85–1.65)	1.15 (0.81–1.65)	0.98	35	63	45	50	31	49	0.90 (0.64–1.28)	1.56 (1.15–2.13)	1.28 (0.88–1.88)	0.03
							***Alcohol***													
GG	133	267	135	306	137	253	1.00 (ref.)	0.98 (0.78–1.25)	1.23 (0.97–1.57)											
GA	150	246	128	261	121	224	1.23 (0.97–1.54)	1.07 (0.84–1.36)	1.21 (0.94–1.55)											
AA	38	46	36	64	37	52	1.70 (1.19–2.42)	1.14 (0.79–1.65)	1.37 (0.95–1.98)	0.41										
GG+GA	283	513	263	567	258	477	1.00 (ref.)	0.92 (0.78–1.09)	1.10 (0.93–1.31)											
AA	38	46	36	64	37	52	1.53 (1.10–2.14)	1.03 (0.73–1.46)	1.24 (0.88–1.75)	0.33										
*NFKB1*/rs28362491							***Red and processed meat***										***Fish***			
I/I	112	238	102	225	106	216	1.00 (ref.)	1.01 (0.78–1.31)	1.06 (0.82–1.37)		99	228	116	214	105	237	1.00 (ref.)	1.09 (0.84–1.41)	0.95 (0.72–1.26)	
I/D+D/D	190	409	203	280	202	351	0.98 (0.78–1.23)	1.46 (1.17–1.83)	1.24 (0.99–1.56)	0.06	207	354	191	328	197	358	1.25 (0.98–1.58)	1.20 (0.94–1.53)	1.13 (0.88–1.44)	0.76
							***Dietary cereal***										***Dietary fibre***			
I/I	105	187	101	212	114	280	1.00 (ref.)	0.87 (0.66–1.15)	0.91 (0.68–1.22)		104	202	112	201	104	276	1.00 (ref.)	1.05 (0.81–1.36)	0.75 (0.58–0.99)	
I/D+D/D	196	302	206	364	193	374	1.10 (0.87–1.39)	1.04 (0.81–1.32)	1.13 (0.85–1.50)	0.76	196	325	194	299	205	416	1.14 (0.90–1.44)	1.16 (0.92–1.46)	0.97 (0.77–1.23)	0.66
							***Fruit***										***Vegetables***			
I/I	113	218	107	216	100	245	1.00 (ref.)	0.96 (0.74–1.25)	0.88 (0.66–1.17)		105	199	114	240	101	240	1.00 (ref.)	1.16 (0.89–1.50)	1.11 (0.83–1.49)	
I/D+D/D	187	308	202	371	206	361	1.15 (0.91–1.45)	1.07 (0.85–1.35)	1.12 (0.87–1.45)	0.70	198	301	190	368	207	371	1.31 (1.04–1.65)	1.19 (0.94–1.52)	1.33 (1.03–1.71)	0.37
							***Alcohol***													
I/I	104	230	111	254	105	195	1.00 (ref.)	1.08 (0.83–1.40)	1.32 (1.01–1.73)											
I/D+D/D	217	329	188	377	190	334	1.45 (1.15–1.83)	1.16 (0.92–1.48)	1.36 (1.08–1.73)	0.09										

^a^ Analysis adjusted for smoking status, alcohol intake, HRT status (women only), BMI, use of NSAID, intake of red and processed meat, and dietary fibre.

^b^ P-value for interaction between polymorphisms and dietary factors for the adjusted estimates

Women: Tertiles of red and processed meat (<74.7773 g, 74.7773 g < and < 102.086 g, >102.086 g), fish (<27.2196 g, 27.2196 g < and < 43.6676 g, > 43.6676 g), dietary fibre (<16.9701 g, 16.9701 g < and < 22.0983 g, > 22.0983 g), cereals (<135.539 g, 135.539 g < and < 190.481 g, > 190.481 g), fruit (<141.858 g, 141.858 g < and < 266.488 g, > 266.488 g), vegetables (<121.870 g, 121.870 g < and < 213.721 g, > 213.721 g), laktose (<7.54871 g, 7.54871 g < and < 17.1355 g, > 17.1355 g), dairy products (<218.144 g, 218.144 g < and < 454.235 g, > 454.235 g), alcohol (<4.31931 g, 4.31931 g < and < 12.9957 g, > 12.9957 g).

Men: Tertiles of red and processed meat (<116.935 g, 116.935 g < and < 159.387 g, >159.387 g), fish (<33.3477 g, 33.3477 g < and < 52.7767 g, > 52.7767 g), dietary fibre (<17.5748 g, 17.5748 g < and < 22.4931 g, > 22.4931 g), cereals (<166.378 g, 166.378 g < and < 233.859 g, > 233.859 g), fruit (<90.9913 g, 90.9913 g < and < 193.509 g, > 193.509 g), vegetables (<105.532 g, 105.532 g < and < 186.459 g, > 186.459 g), laktose (<7.93777 g, 7.93777 g < and < 17.2082 g, > 17.2082 g), dairy products (<217.360 g, 217.360 g < and < 461.449 g, > 461.449 g), alcohol (<14.4960 g, 14.4960 g < and < 37.1134 g, > 37.1134 g).

There was no interaction between NSAID use or smoking status and the studied genotypes (S3 and S4 Tables). Among non-smokers (S4 Table), however, the *TLR4*/rs5030728 polymorphism demonstrated gene-dose effect which is comparable with the results seen among participants with low intake of alcohol (Table 3). In a model where the risk of CRC was inferred per 25 g intake of meat per day subdivided by NSAID use, the risk of CRC by meat intake increased in a dose-dependent manner among variant allele carriers of *TLR4*/rs5030728 in the absence of NSAID use, but not among NSAID-users (S5 Table). Thus, meat intake was not associated with risk among homozygous carriers of the wild type allele, whereas meat intake was associated with a 4% increased risk per 25 g meat/day (95%CI: 1.00–1.09) among heterozygotes and 11% increased risk among homozygous variant allele carriers (95%CI: 1.02–1.22). However, there were no statistically significant interactions (S5 Table).

## Discussion

In the present study, we found that homozygous variant carriage of *TLR4*/rs5030728 and variant carriage of the *NFKB1*/rs28362491 polymorphism were associated with increased risk of CRC, but not after correction for multiple testing. We only found weak interactions with a few dietary factors and, thus, we were not able to reproduce the previously found interaction between the *NFKB1*/rs28362491 polymorphism and meat intake. The lack of association between *TLR4*/rs4986790 and CRC found in the present study could possibly be due to the very low variant allele frequency in the Danish population. Only one person was homozygous variant allele carrier. We therefore cannot exclude that the functional effect of this SNP affects colorectal carcinogenesis.

The *NFKB1*/rs28362491 polymorphism has rather consistently been associated with CRC risk [30–32], and to some extend also IBD [29,63,64]. As previously described [65,66], *NFKB1* encodes the p50/p105 subunits of the transcription factor NF-κB. NF-κB consists of homo- or heterodimers of a number of different subunits p65, p50, p105, C-rel and relB [67,68] and the combination determines target gene specificity. As a p65/p50 heterodimer, the complex is pro-inflammatory [68], whereas the p50 homodimer has anti-inflammatory properties [65,67,69,70]. The relative abundance of p50/p65 heterodimers and p50 homodimers will therefore determine the magnitude of inflammation by balancing the pro-inflammatory and anti-inflammatory response [67]. The *NFKB1*/rs28362491 polymorphism generates a deletion of four nucleotides in the promoter region causing lowered transcription levels and consequently partial depletion of p50 [63]. In agreement with this, it was found that the mRNA levels of *NFKB1* were lower in colon biopsies of healthy tissue from homozygous del-carriers compared to heterozygotes [71]. This disfavours the anti-inflammatory response since the formation of the pro-inflammatory p65/p50 heterodimer depends on the concentration of p50, whereas the formation of the anti-inflammatory p50 homodimer depends on the concentration of p50 squared [65].

The *TLR4*/rs5030728 polymorphism has not yet been linked to CRC [20] and its function is unknown [72]. *TLR4*/rs4986790, which has been associated with IBD and CRC, is tightly linked with *TLR4*/rs5030728. It is therefore not clear which of the two polymorphisms is the biologically relevant one. However, our results indicate that carriage of *TLR4*/rs4986790 was not associated with risk of CRC, whereas carriage of *TLR4*/rs5030728 was associated with risk. This suggests that the risk conferred by *TLR4*/rs5030728 carriage was not caused by linkage with *TLR4*/rs4986790. Interestingly, variant carriage of the *TLR4*/rs5030728 A-allele has been associated with beneficial response to anti-TNF therapy among patients with IBD [73], implying that these patients may have a higher baseline activity or expression of TLR4.

The two *TLR4* SNP are present on several commonly used GWAS arrays (https://www.broadinstitute.org/mpg/snap/ldsearch.php) whereas the ins/del *NFKB1*/rs28362491 polymorphism is not monitored linkage in GWAS [65]. None of the two TLR4 SNPs were associated with CRC in GWAS. However, our main focus was to search for gene-environment interactions, rather than identifying loci with strong associations to CRC. Gene-environment interactions are rarely assessed in GWAS.

We did not find any strong indications of gene-environment interactions. For variant carriers of *NFKB1*/rs28362491 and homozygous A-allele carriers of *TLR4*/rs5030728, risk of CRC among low meat consumers was lower compared to medium and high meat consumers, who had risk estimates between 1.24 and 1.70 indicating a stronger role of meat in colorectal carcinogenesis among subjects with genetically determined high inflammatory response. Alternatively, meat intake covaries with other life style factors that *per se* induce an inflammatory response that we have not been able to adjust for.

The found interaction with vegetables and *TLR4*/rs5030728 in the present study is not directly interpretable and could be due to small groups in the tertiles. However, vegetables seemed to slightly increase the risk of CRC in the present study, which should be addressed in other prospective studies.

We had limited statistical power to detect gene-environment interactions. However, the prospective study design used in this study is well suited for gene-environment interaction analyses due to the collection of dietary and life style factors before diagnosis, eliminating the risk of recall bias. Changing in dietary and life style habits during follow-up is, however, possible, but is not expected to result in differential misclassification between cases and the comparison group. In addition, the present study group is homogenous consisting of Danes and two of the studied polymorphisms have high allele frequencies. Using the present study group, we have previously found gene-environment interactions between diet and *IL10* rs3024505 (P_int_;meat = 0.04, fish = 0.007, fibre = 0.0008, vegetables = 0.0005), *IL1B* C-3737T (P_int_; NSAID use = 0.040), *PTGS2* G-765C (P_int_; meat = 0.006, fibre = 0.0003, fruit 0.004), and *PTGS2* T8473C (P_int_; fruit = 0.03) and *PTGS2* A-1195G (P_int_;fibre 0.020 and current smoking = 0.046) [27]. We adjusted risk estimates for suspected risk factors and carefully selected the polymorphisms based on function and/or previously findings on association with dietary factors, CRC or IBD. However, none of the analyses withstood adjusting for multiple testing. Thus, we cannot rule out that our findings are due to chance and they should therefore not be considered as significant associations.

In conclusion, this study was not able to demonstrate associations between the studied polymorphisms in the inflammatory mediator genes *NFKB1* and *TLR4* as none of the found associations withstood adjustment for multiple comparisons. We found no strong gene-environment interactions between the examined polymorphisms and diet and life style factors in relation to CRC risk.

## Supporting Information

S1 Table IRR for CRC in relation to combinations of *NFKB1*/rs2836249 and *TLR4*/rs5030728 genotypes. (DOCX)

S2 Table Interaction between dietary factors and the studied polymorphisms in relation to CRC risk. (DOCX)

S3 Table Interaction between NSAID use and the studied polymorphisms in relation to CRC risk. (DOCX)

S4 Table Interaction between smoking status and the studied polymorphisms in relation to risk of CRC. (DOCX)

S5 Table Interaction between NSAID use and the studied polymorphisms per 25 g red and processed meat intake per day in relation to CRC risk. (DOCX)
