# Supplementary material for: Polymorphisms in NFKB1 and TLR4 and Interaction with Dietary and Life Style Factors in Relation to Colorectal Cancer in a Danish Prospective Case-Cohort Study
Source: PLoS One. 2015 Feb 23;10(2):e0116394. doi: 10.1371/journal.pone.0116394 (PMC4337910; doi:10.1371/journal.pone.0116394)
Supplement: S1 Table — (DOCX) [file pone.0116394.s001.docx]

**Table S1. IRR for CRC in relation to combinations of *NFKB1*/rs2836249 and *TLR4*/rs5030728 genotypes.**

| Genotype | NFKB1/rs2836249 | | NFKB1/rs2836249 | | NFKB1/rs2836249 | | P-value^c^ |
| --- | --- | --- | --- | --- | --- | --- | --- |
|  | Ins/Ins  n_cases_/ n_controls_ | Ins/Del+Del/Del  n_case_/ n_controls_ | Ins/Ins  IRR (95% CI)^a^ | Ins/Del+Del/Del  IRR (95% CI) ^a^ | Ins/Ins  IRR (95% CI)^b^ | Ins/Del+Del/Del  IRR (95% CI)^b^ |  |
| *TLR4*/rs5030728  GG  GA  AA  GG+GA  AA | 145/335  131/281  44/63  276/616  44/63 | 260/491  268/450  67/99  528/941  67/99 | 1.00 (ref.)  1.11 (0.88-1.40)  1.53 (1.10-2.14)  1.00 (ref.)  1.46 (1.07-2.00) | 1.22 (1.00-1.49)  1.32 (1.08-1.61)  1.43 (1.07-1.91)  1.21 (1.05-1.40)  1.37 (1.04-1.78) | 1.00 (ref.)  1.12 (0.89-1.41)  1.50 (1.08-2.10)  1.00 (ref.)  1.43 (1.04-1.96) | 1.22 (1.00-1.49)  1.33 (1.09-1.62)  1.44 (1.08-1.93)  1.21 (1.05-1.40)  1.37 (1.05-1.80) | 0.55  0.27 |

^a^ Crude – adjusted for age and sex.

^b^ In addition, adjusted for smoking status, alcohol intake, HRT status (women only), BMI, use of NSAID, intake of red and processed meat, and dietary fibre.

^c^ P-value for interaction for the adjusted risk estimates.
