# Supplementary material for: Polymorphisms in NFKB1 and TLR4 and Interaction with Dietary and Life Style Factors in Relation to Colorectal Cancer in a Danish Prospective Case-Cohort Study
Source: PLoS One. 2015 Feb 23;10(2):e0116394. doi: 10.1371/journal.pone.0116394 (PMC4337910; doi:10.1371/journal.pone.0116394)
Supplement: S2 Table — (DOCX) [file pone.0116394.s002.docx]

**Table S2. Interaction between dietary factors and the studied polymorphisms in relation to CRC risk.**

|  |  | IRR^a^ (95% CI) | *P*-value^b^ | IRR^a^ (95% CI) | *P*-value^b^ | IRR^a^ (95% CI) | *P*-value^b^ | IRR^a^ (95% CI) | *P*-value^b^ |
| --- | --- | --- | --- | --- | --- | --- | --- | --- | --- |
|  |  | ***Red and processed meat per 25 g/day*** | | ***Fish per 25 g/day*** | | ***Dietary cereal per 50 g/day*** | | ***Dietary fibre per 10 g/day*** | |
| *TLR4* | rs4986790  AA  GA+GG | 1.03 (1.00-1.06)  0.99 (0.87-1.13) | 0.56 | 0.96 (0.89-1.03)  0.95 (0.75-1.20) | 0.96 | 1.02 (0.95-1.10)  1.06 (0.91-1.25) | 0.61 | 0.88 (0.80-0.97)  0.85 (0.60-1.21) | 0.86 |
|  | rs5030728  GG  GA+AA | 1.01 (0.97-1.06)  1.04 (1.01-1.08) | 0.29 | 1.00 (0.90-1.10)  0.92 (0.85-1.01) | 0.24 | 1.05 (0.96-1.14)  1.01 (0.93-1.10) | 0.44 | 0.88 (0.77-1.00)  0.88 (0.77-1.00) | 0.96 |
| *NFKB1* | rs28362491  Ins/Ins  Ins/Del+Del/Del | 1.01 (0.97-1.06)  1.04 (1.00-1.07) | 0.46 | 0.95 (0.86-1.05)  0.96 (0.89-1.04) | 0.84 | 1.02 (0.92-1.12)  1.04 (0.96-1.12) | 0.63 | 0.89 (0.76-1.04)  0.87 (0.77-0.98) | 0.83 |
|  |  | ***Fruit per 50 g/day*** | | ***Vegetables per 50 g/day*** | | ***Alcohol per 10 g/day^c^*** | |  | |
| *TLR4* | rs4986790  AA  GA+GG | 0.98 (0.96-1.01)  0.96 (0.90-1.03) | 0.58 | 1.02 (0.98-1.07)  0.96 (0.85-1.09) | 0.36 | 1.04 (1.01-1.07)  0.98 (0.88-1.09) | 0.29 |  |  |
|  | rs5030728  GG  GA+AA | 0.97 (0.94-1.01)  0.98 (0.95-1.02) | 0.63 | 1.02 (0.96-1.07)  1.02 (0.97-1.07) | 0.91 | 1.05 (1.01-1.10)  1.02 (0.98-1.06) | 0.31 |  |  |
| *NFKB1* | rs28362491  Ins/Ins  Ins/Del+Del/Del | 0.98 (0.94-1.02)  0.98 (0.95-1.01) | 0.97 | 1.03 (0.97-1.09)  1.01 (0.96-1.06) | 0.58 | 1.07 (1.02-1.12)  1.02 (0.98-1.05) | 0.10 |  |  |

^a^ Adjusted for age, sex, smoking status, alcohol, HRT status (women only), BMI, use of NSAID, intake of red and processed meat, and dietary fibre.

^b^ P-value for interaction for adjusted risk estimates.

^c^ Among current drinkers only.
