# Supplementary material for: Polymorphisms in NFKB1 and TLR4 and Interaction with Dietary and Life Style Factors in Relation to Colorectal Cancer in a Danish Prospective Case-Cohort Study
Source: PLoS One. 2015 Feb 23;10(2):e0116394. doi: 10.1371/journal.pone.0116394 (PMC4337910; doi:10.1371/journal.pone.0116394)
Supplement: S3 Table — (DOCX) [file pone.0116394.s003.docx]

**Table S3. Interaction between NSAID use and the studied polymorphisms in relation to CRC risk.**

|  |  | NSAID use  n_cases_/n_subcohorte_ | | NSAID use  IRR (95% CI)^a^ | | NSAID use  IRR (95% CI)^b^ | | P-value^c^ |
| --- | --- | --- | --- | --- | --- | --- | --- | --- |
|  |  | No | Yes | No | Yes | No | Yes |  |
| *TLR4* | rs4986790  AA  GA+GG | 604/1051  51/100 | 261/500  25/42 | 1.00 (ref.)  0.95 (0.72-1.27) | 0.98 (0.85-1.13)  0.98 (0.66-1.43) | 1.00 (ref.)  0.96 (0.72-1.28) | 0.97 (0.84-1.12)  0.95 (0.64-1.42) | 0.94 |
|  | rs5030728  GG  GA  AA  GG+GA  AA | 556/556  291/489  67/106  847/1649  67/106 | 122/256  117/233  47/53  239/489  47/53 | 1.00 (ref.)  1.10 (0.94-1.29)  1.15 (0.88-1.50)  1.00 (ref.)  1.10 (0.85-1.42) | 0.95 (0.77-1.17)  1.01 (0.92-1.25)  1.50 (1.11-2.03)  0.94 (0.81-1.09)  1.43 (1.07-1.92) | 1.00 (ref.)  1.11 (0.94-1.30)  1.14 (0.88-1.49)  1.00 (ref.)  1.09 (0.84-1.40) | 0.95 (0.77-1.17)  1.00 (0.81-1.24)  1.47 (1.08-2.00)  0.93 (0.80-1.07)  1.40 (1.04-1.88) | 0.26  0.11 |
| *NFKB1* | rs28362491  Ins/Ins  Ins/Del+Del/Del | 236/455  419/696 | 93/215  240/327 | 1.00 (ref.)  1.12 (0.96-1.32) | 0.90 (0.71-1.14)  1.16 (0.96-1.40) | 1.00 (ref.)  1.13 (0.96-1.32) | 0.89 (0.70-1.13)  1.15 (0.95-1.38) | 0.37 |

^a^ Crude – adjusted for age and sex.

^b^ In addition, adjusted for smoking status, alcohol, HRT status (women only), BMI, intake of red and processed meat, and dietary fibre.

^c^ P-value for interaction for the adjusted estimates.
