# Supplementary material for: Polymorphisms in NFKB1 and TLR4 and Interaction with Dietary and Life Style Factors in Relation to Colorectal Cancer in a Danish Prospective Case-Cohort Study
Source: PLoS One. 2015 Feb 23;10(2):e0116394. doi: 10.1371/journal.pone.0116394 (PMC4337910; doi:10.1371/journal.pone.0116394)
Supplement: S4 Table — (DOCX) [file pone.0116394.s004.docx]

**Table S4. Interaction between smoking status and the studied polymorphisms in relation to risk of CRC .**

| Gene | Genotype | Never smokers | Past smokers | Current smokers | Never smokers | Past smokers | Current smokers | Never smokers | Past smokers | Current smokers | p-value^c^ |
| --- | --- | --- | --- | --- | --- | --- | --- | --- | --- | --- | --- |
|  |  | n_cases_/n_sub-cohort_ | n_cases_/n_sub-cohort_ | n_cases_/n_sub-cohort_ | IRR (95%CI)^a^ | IRR (95%CI)^a^ | IRR (95%CI)^a^ | IRR (95%CI)^b^ | IRR (95%CI)^b^ | IRR (95%CI)^b^ |  |
| *TLR4* | rs4986790  AA  GA+GG | 249/524  25/48 | 258/470  22/43 | 332/583  29/51 | 1.00 (ref.)  1.08 (0.72-1.62) | 1.05 (0.88-1.25)  0.99 (0.64-1.52) | 1.15 (0.98-1.36)  1.11 (0.76-1.61) | 1.00 (ref.)  1.04 (0.69-1.58) | 1.05 (0.88-1.25)  0.99 (0.63-1.53) | 1.11 (0.94-1.31)  1.11 (0.76-1.62) | 0.94 |
|  | rs5030728  GG  GA  AA  GG+GA  AA | 113/279  124/237  37/56  237/516  37/56 | 130/251  114/216  36/46  244/467  36/46 | 162/296  161/278  38/60  323/574  38/60 | 1.00 (ref.)  1.29 (1.00-1.66)  1.56 (1.07-2.29)  1.00 (ref.)  1.38 (0.97-1.98) | 1.17 (0.91-1.50)  1.19 (0.92-1.54)  1.53 (1.05-2.24)  1.04 (0.87-1.25)  1.36 (0.95-1.93) | 1.31 (1.03-1.67)  1.35 (1.07-1.71)  1.46 (1.02-2.09)  1.18 (1.00-1.39)  1.29 (0.92-1.80) | 1.00 (ref.)  1.30 (1.01-1.68)  1.59 (1.09-2.31)  1.00 (ref.)  1.39 (0.98-1.99) | 1.18 (0.92-1.51)  1.22 (0.94-1.58)  1.53 (1.05-2.25)  1.05 (0.88-1.26)  1.35 (0.94-1.93) | 1.28 (1.01-1.63)  1.32 (1.04-1.67)  1.40 (0.98-2.01)  1.14 (0.96-1.36)  1.23 (0.88-1.73) | 0.50  0.57 |
| *NFKB1* | rs28362491  Ins/Ins  Ins/Del+Del/Del | 97/221  177/351 | 85/212  195/301 | 138/246  223/388 | 1.00 (ref.)  1.17 (0.91-1.49) | 0.91 (0.68-1.21)  1.29 (1.01-1.64) | 1.24 (0.96-1.60)  1.27 (1.00-1.61) | 1.00 (ref.)  1.17 (0.92-1.50) | 0.91 (0.68-1.22)  1.30 (1.02-1.66) | 1.21 (0.94-1.57)  1.24 (0.97-1.57) | 0.13 |

^a^ Crude – adjusted for age and sex.

^b^ In addition, adjusted for alcohol intake, HRT status (women only), BMI, use of NSAID, intake of red and processed meat, and dietary fibre.

^c^ P-value for interaction for adjusted risk estimates.
