# Supplementary material for: Polymorphisms in NFKB1 and TLR4 and Interaction with Dietary and Life Style Factors in Relation to Colorectal Cancer in a Danish Prospective Case-Cohort Study
Source: PLoS One. 2015 Feb 23;10(2):e0116394. doi: 10.1371/journal.pone.0116394 (PMC4337910; doi:10.1371/journal.pone.0116394)
Supplement: S5 Table — (DOCX) [file pone.0116394.s005.docx]

**Table S5. Interaction between NSAID use and the studied polymorphisms per 25 g red and processed meat intake per day in relation to CRC risk.**

|  |  | NSAID use  n_cases_/n_subcohorte_ | | NSAID use  IRR (95% CI)^a^ | | P-value^b^ |
| --- | --- | --- | --- | --- | --- | --- |
|  |  | No | Yes | No | Yes |  |
| *TLR4* | rs4986790  AA  GA+GG | 604/1051  51/100 | 261/500  25/42 | 1.03 (1.00-1.07)  1.03 (0.89-1.18) | 1.03 (0.98-1.08)  0.89 (0.65-1.22) | 0.84 |
|  | rs5030728  GG  GA  AA  GG+GA  AA | 556/556  291/489  67/106  847/1649  67/106 | 122/256  117/233  47/53  239/489  47/53 | 1.00 (0.95-1.05)  1.04 (1.00-1.09)  1.11 (1.02-1.22)  1.02 (0.99-1.06)  1.11 (1.02-1.22) | 1.04 (0.98-1.10)  1.02 (0.95-1.10)  0.98 (0.85-1.12)  1.03 (0.98-1.08)  0.98 (0.85-1.12) | 0.39  0.30 |
| *NFKB1* | rs28362491  Ins/Ins  Ins/Del+Del/Del | 236/455  419/696 | 93/215  240/327 | 1.00 (0.94-1.05)  1.05 (1.01-1.09) | 1.06 (0.98-1.16)  1.00 (0.95-1.06) | 0.27 |

^a^ Adjusted for age, sex, smoking status, alcohol intake, HRT status (women only), BMI, and dietary fibre.

^b^ P-value for interaction for the adjusted estimates.
